# Effect of CeO_2_ Content on Microstructure and Properties of SiCp/Al-Si Composites Prepared by Powder Metallurgy

**DOI:** 10.3390/ma14164685

**Published:** 2021-08-19

**Authors:** Xuedan Dong, Aiqin Wang, Jingpei Xie, Pengfei Zhu

**Affiliations:** 1 School of Materials Science and Engineering, Henan University of Science and Technology, Luoyang 471023, China; dongxd98@163.com (X.D.); xiejp@haust.edu.cn (J.X.); zhoukou19870708@163.com (P.Z.); 2 Collaborative Innovation Center of Nonferrous Metals, Luoyang 471023, China

**Keywords:** powder metallurgy, CeO_2_, microstructure, tensile properties

## Abstract

The effect of CeO_2_ content on the microstructure and properties of SiCp/Al-Si composites prepared by powder metallurgy was studied, and the mechanism of CeO_2_ in composites was deeply analyzed. The results show that the addition of the appropriate amount of CeO_2_ can refine the Si particles and improve the tensile properties of the SiCp/Al-Si composites. As the CeO_2_ content increases from 0 to 0.4 vol%, the particle size of the Si phase shows a tendency to decrease first and then increase, while the tensile strength, yield strength, and elongation of the composites show a trend of first increasing and then decreasing. When the CeO_2_ content is 0.2 vol%, the refining effect of CeO_2_ and the tensile properties of composites are the best. The fracture mode of SiCp/Al-Si composites with a rare earth addition is a mixed fracture. There are three main mechanisms for CeO_2_ in SiCp/Al-Si composites. One is when CeO_2_ serves as the nucleation substrate of Si phase to refine Si particles. The second is when CeO_2_ reacts with the alloying elements in the aluminum matrix to form a new phase, CeCu_2_Si_2_, which can not only play a role of dispersion strengthening, but also improve the bonding strength between Al matrix and Si particles. The third is the pinning effect of CeO_2_ and CeCu_2_Si_2_ particles on grain boundaries or phase boundaries to refine aluminum grains.

## 1. Introduction

Al-Si alloys are widely used as lightweight materials in aerospace, automotive parts, and other fields due to their excellent casting properties, high strength, and strong corrosion resistance [1,2]. SiCp has the characteristics of isotropy, low expansion coefficient, and high strength, which are ideal reinforcing materials for preparing metal matrix composites [3,4]. SiCp/Al-Si composites combine the advantages of SiC and Al-Si alloys in strength, expansion, and wear resistance [5,6]. Li et al. [7] studied the effect of the SiCp orientation anisotropy on the tensile properties of spray-formed SiCp/Al-Si composites compared with that of the matrix alloy without reinforcement particles. The results showed that the addition of SiCp improved the tensile properties of the Al-Si alloy. Zhou et al. [8] fabricated SiCp/Al composites based on different matrix elements, Mg and Si, content by pressureless infiltration. The results showed that adding the Mg element to the matrix increased the coefficient of thermal expansion (CTE) of SiCp/Al composites and adding the Si element to the matrix could control the formation of harmful interface products A1_4_C_3_.

For Al-Si cast alloys, the size and morphology of silicon play a decisive role in the properties of the alloys [9]. The eutectic silicon in the unmodified alloy is usually thick and lamellar, which tends to produce stress concentration at the tip of eutectic silicon, thereby reducing the elongation of the alloy. Rare earth elements are often used as modifiers because of their high chemical activity. Among them, Ce, La, and Y are the most commonly used rare earth elements in aluminum alloy [10,11]. In the case of Al-Si alloys, tiny amounts of rare earth elements can transform the second phase (Si phase) from a thick plate to a fine fibrous shape. The particle size of the Si phase becomes smaller after further heat treatment (solution aging), and the performance of the alloy has been greatly improved [12,13]. At present, rare earth is often introduced into Al-Si matrix composites in the form of rare earth compounds. In the Al-Si matrix composites prepared by the casting method, rare earth is usually added in the form of ReH_2_, the Re-Al series master alloy, and ReO_2_ [14,15]. Sharma et al. [16] prepared the Al-11Si-20Cu brazing alloy containing La_2_O_3_ by the electromagnetic induction casting process and found that the addition of La_2_O_3_ significantly improved the filler brazeability, microhardness, joint tensile shear strength, and elongation of the composites. Elgallad et al. [17] found that adding rare earth elements, La or Ce, to the Al-Si alloy increased the melting temperature of the alloy and caused partial modification of the eutectic Si particles. Liu et al. [18] added different contents of CeO_2_ and Y_2_O_3_ (2 vol% and 3 vol%) to Ti/Al_2_O_3_ composites prepared by vacuum hot pressing sintering to restrain the interface reaction. The results indicated that rare earth oxides effectively reduced the thicknesses of the reaction layers and improve the performance of the composites.

Powder metallurgy is one of the earliest solid forming methods for preparing particle reinforced aluminum matrix composites, and the process is relatively mature [19,20,21]. However, the research on particle reinforced aluminum matrix composites with rare earth mostly focuses on the preparation of composites by the liquid phase method. There are few reports on the application of rare earth added in Al-Si matrix composites prepared by powder metallurgy, and less attention has been paid to the mechanism of rare earth in Al-Si matrix composites. In this paper, SiCp/Al-Si composites with different CeO_2_ volume fraction were prepared by the powder metallurgy method. The effect of CeO_2_ content on the microstructure and properties of the composites was investigated. Additionally, the mechanism of CeO_2_ in the SiCp/Al-Si composite was also explored.

## 2. Materials and Methods

In this paper, SiCp/Al-Si composites with different CeO_2_ contents prepared by powder metallurgy were used as the research object. Al-12Si alloy powder with a particle size of 7 µm was used as the matrix. Table 1 shows its chemical composition. SiCp, with an average particle size of 5 µm and a volume fraction of 20%, was selected as reinforcement. The additive used in the experiments was CeO_2_ powder, with a particle size of about 1~20 μm, and its addition amount was 0, 0.1 vol%, 0.2 vol%, 0.3 vol%, and 0.4 vol%, respectively. The experimental preparation process was powder mixing, cold pressing, sintering, hot extrusion, and heat treatment. The specific technological parameters were as follows: QM-BP planetary ball mill (Shenhua Biotechnology, Guangzhou, China) was used for dry mixing, the grinding ball was a stainless-steel ball, the dispersant was stearic acid, the ball material ratio was 4:1 (mass ratio), the ball mill speed was 240 r/min, and the mixing time was 6 h. The billet, with a diameter of 78 mm, was then cold-pressed in a hydraulic machine (Zhengxi, Chengdu, China) with a pressure of 500 Mpa. After that, the billet was heated in a tube furnace (NBD, Zhengzhou, China) with a sintering temperature of 530 °C, a sintering time of 4 h, a heating rate of 2 °C/min, and a protective atmosphere of nitrogen (N_2_). The billet was then hot extruded into the bar, with an extrusion ratio of 15.6:1 and an extrusion speed of 1 mm/s at about 500 °C. Finally, the hot extruded samples were annealed at 300 °C for 3 h, solution treated at 510 °C for 2 h, water quenched, and then immediately aged at 170 °C for 5 h.

**Figure 1:**
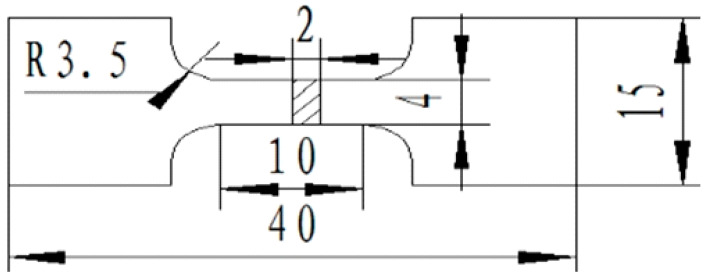
Schematic diagram of tensile sample (mm).

The microstructures of the samples were observed by JSM-5610LV scanning electron microscopy (SEM, JEOL, Tokyo, Japan), with energy dispersive spectroscopy (EDS, Kevex, Texas, TX, USA) and transmission electron microscopy (TEM, JEOL, Tokyo, Japan). The preparation method of the TEM samples was to first cut the material into a thin slice with a thickness of 0.3 mm and then manually polish the slice to a thickness of about 50 μm with sandpaper. Next, it was punched into thin slices with a diameter of 3 mm and finally thinned on the Gatan-691 ion thinner. The tensile properties of the composites were tested by the Shimazu AG-1250kN precision universal tensile testing machine (Shimazu, Tokyo, Japan), with an initial strain rate of 1.1 × 10^−3^ s^−1^ at room temperature. The size of tensile sample was shown in Figure 1, and the tensile results were the average values of three samples for each tested condition.

## 3. Results and Discussion

### 3.1. Effect of CeO_2_ Content on Microstructure of SiCp/Al-Si Composites

**Figure 2:**
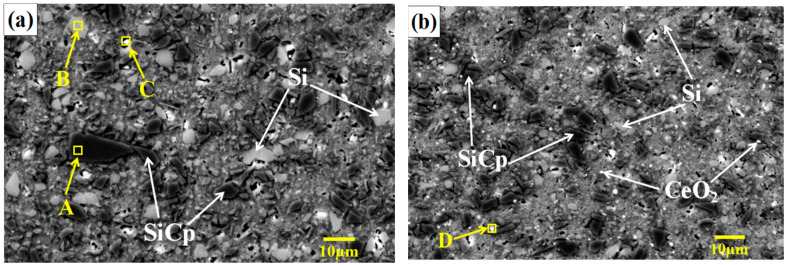

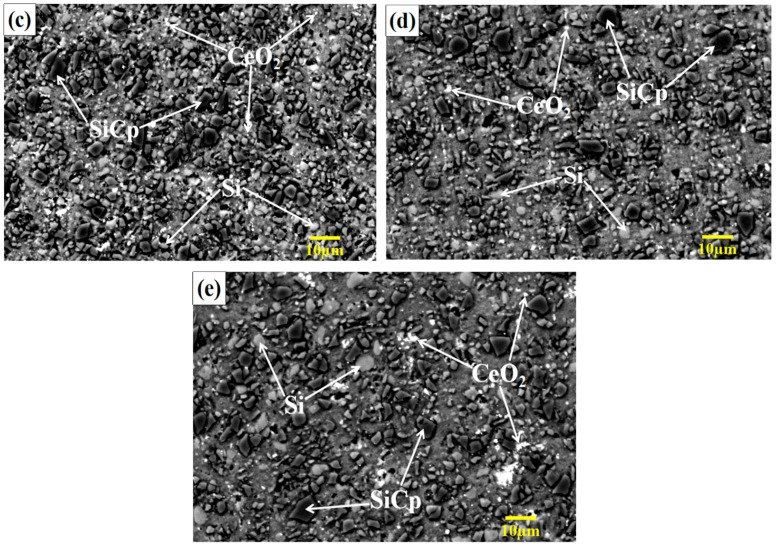
The SEM images of SiCp/Al-Si composites with different CeO_2_ volume fraction: (**a**) 0; (**b**) 0.1 vol%; (**c**) 0.2 vol%; (**d**) 0.3 vol%; (**e**) 0.4 vol%.

**Figure 3:**
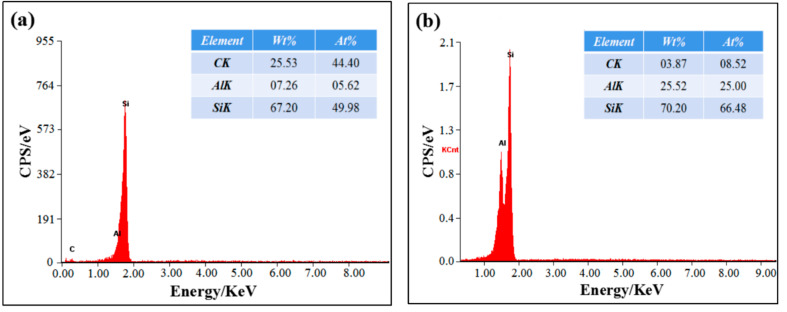

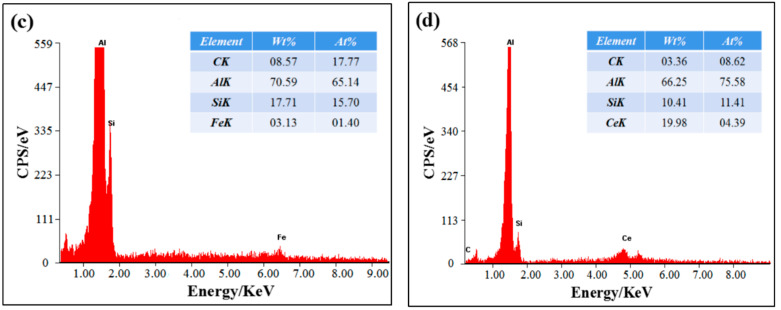
The energy spectrum diagram of SiCp/Al-Si composites with different CeO_2_ volume fraction: (**a**) the EDS analysis of region A in Figure 2a; (**b**) the EDS analysis of region B in Figure 2a; (**c**) the EDS analysis of region C in Figure 2a; (**d**) the EDS analysis of region D in Figure 2b.

Figure 2 shows the microstructure of SiCp/Al-Si composites with different CeO_2_ volume fraction. According to the energy spectrum analysis in Figure 3, the black phase with irregular shapes and sharp edges in Figure 2 is SiC. The gray phase with regular shapes, mostly round and elliptical, and smooth edges was Si. The white, spotty, or flocculent phases were ferric or cerium-containing phases. The Fe element in the Fe phase may have come from the impurity elements in the Al matrix or have been mixed in ball milling.

**Figure 4:**
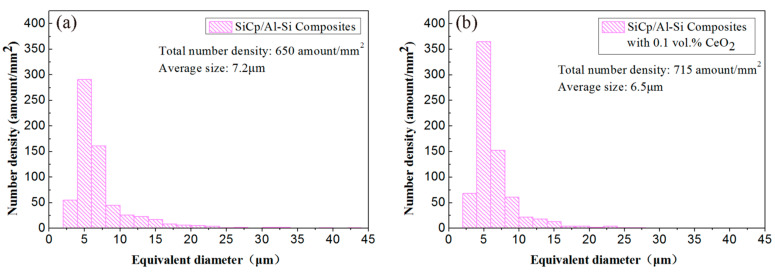

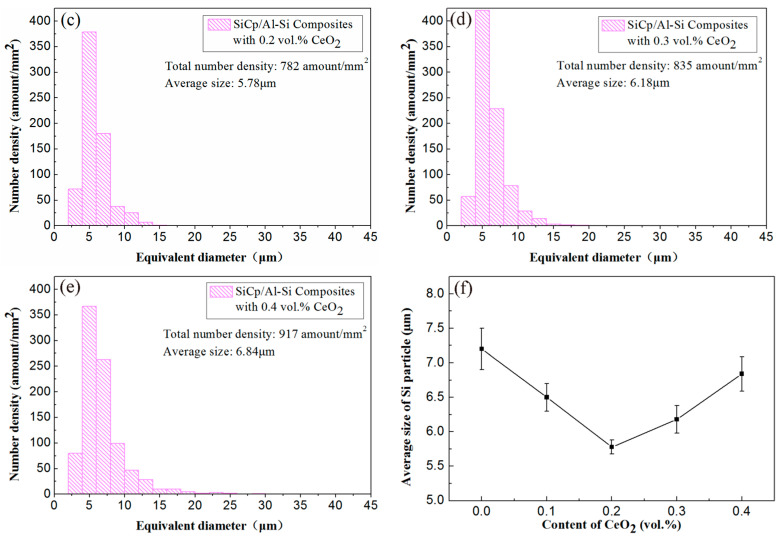
The number density distribution histogram and the average size of precipitated Si particles in SiCp/Al-Si composites with different CeO_2_ contents: (**a**) 0; (**b**) 0.1 vol%; (**c**) 0.2 vol%; (**d**) 0.3 vol%; (**e**) 0.4 vol%; (**f**) the average size of precipitated Si particles.

Figure 4 shows the number density distribution histogram and the average size of the Si phase in the composites with different CeO_2_ contents. According to the normal distribution, as the size of the Si phase particles increases, the number density of the particles decreases gradually. Combining Figure 2 and Figure 4, it can be seen that, when CeO_2_ was not added, the precipitated Si particles were thick, unevenly distributed, and seriously agglomerated. When the addition amount of CeO_2_ was low, the size of Si phase decreased significantly, and the distribution uniformity increased with the increase of CeO_2_ content. When the content of CeO_2_ was 0.2 vol%, the size of Si phase was the smallest and the distribution was the most uniform. At this time, the white cerium phase was mostly punctate and evenly distributed. When the CeO_2_ content exceeded 0.2 vol%, the white cerium phase agglomerated and became larger in size. In addition, the size of the Si phase gradually increased.

The addition of CeO_2_ can refine the Si phase and make its distribution more uniform. Firstly, CeO_2_ can be used as the heterogeneous nucleation site of Si phase, which increases the number density of Si phase, thus refining the Si particle. When the content of CeO_2_ was low, CeO_2_ could be used as the nucleation substrate to refine Si phase effectively. When the CeO_2_ content was 0.1 vol%, the content of CeO_2_ was insufficient and the refining was not sufficient. When the CeO_2_ content was 0.2 vol%, the addition amount of CeO_2_ was appropriate and the refining effect was most obvious. When the CeO_2_ content exceeded 0.2 vol%, CeO_2_ agglomerated, thereby deteriorating the refining effect. When the CeO_2_ content was 0.4 vol%, the silicon phase was coarse and agglomerated, which was almost the same as the structure without CeO_2_, and the refinement effect was the worst. Secondly, the addition of an appropriate amount of CeO_2_ can make the Si phase distribution more uniform. This may be because the dispersed rare earths can provide a large base for the growth of Si phase and attract a large number of Si elements, so that the Si element required for the agglomeration of Si phase is insufficient, and thus reduce the agglomeration tendency of Si phase, and the uniformity of Si phase distribution could be improved accordingly.

### 3.2. TEM Analysis of SiCp/Al-Si Composites

**Figure 5:**
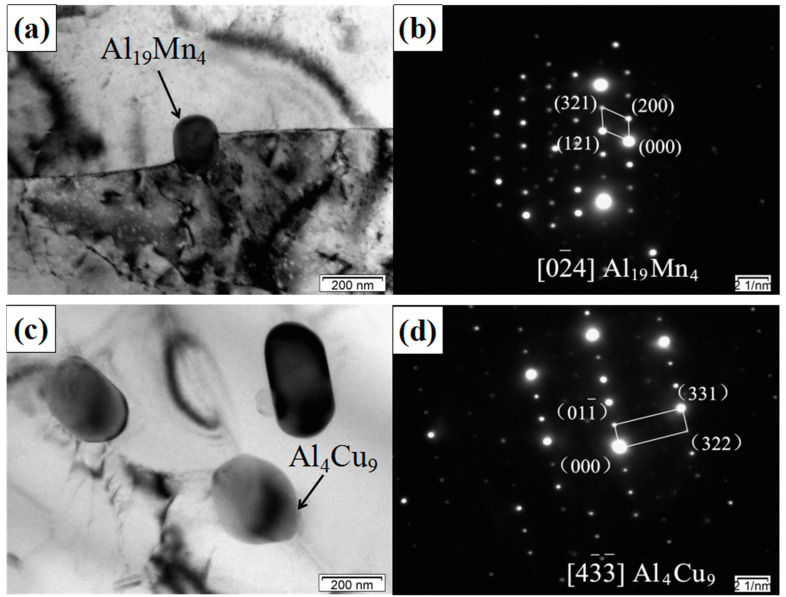

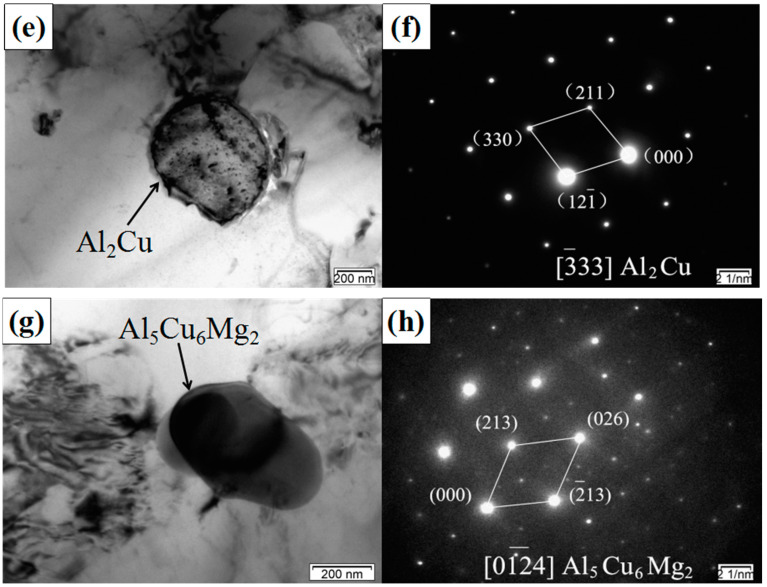
TEM images of SiCp/Al-Si composites without CeO_2_: (**a**) the precipitated phase Al_19_Mn_4_; (**b**) diffraction patterns of Al_19_Mn_4_; (**c**) the precipitated phase Al_4_Cu_9_; (**d**) diffraction patterns of Al_4_Cu_9_; (**e**) the precipitated phase Al_2_Cu; (**f**) diffraction patterns of Al_2_Cu; (**g**) the precipitated phase Al_5_Cu_6_Mg_2_; (**h**) diffraction patterns of Al_5_Cu_6_Mg_2_.

The matrix Al-12Si-Cu-Mg is a typical heat-treatable aluminum alloy, in which the main precipitates are Mg_2_Si, Al_2_Cu, Al_2_CuMg, Al_5_Cu_2_Mg_8_Si_6_, and Al(Si, Fe)Mn phases [22,23]. Figure 5 shows the TEM images and corresponding selected-area electron diffraction (SAED) patterns of SiCp/Al-Si composites without CeO_2_ after heat treatment. Some dispersed and coarse second-phase particles could be seen in the SiCp/Al-Si composites prepared by powder metallurgy, as shown in Figure 5. The results of SAED patterns show that these large round and rod-like particles may have been Al_4_Cu_9_, Al_2_Cu, Al_19_Mn_4_, or Al_5_Cu_6_Mg_2_, and not Mg_2_Si, Al_5_Cu_2_Mg_8_Si_6_, or Al(Si, Fe)Mn dispersoids. It can be seen that, in the SiCp/Al-Si composites, there were more compounds formed between Al-Cu, Al-Cu-Mg, and Al-Mn, while the particles containing Si or Fe were not found. This may be due to the sintering temperature (530) of the composites prepared by powder metallurgy, which was lower than the molten temperature of the cast Al-Si-Cu-Mg alloy. The atom diffusion ability of the composites was weaker than that of the cast alloy. In addition, affected by heat, vacancy concentration, internal stress, and other factors, it is more difficult for Si and Fe elements to combine with other elements to form compounds compared with Al, Cu, Mg, and Mn in the low-temperature aging process.

**Figure 6:**
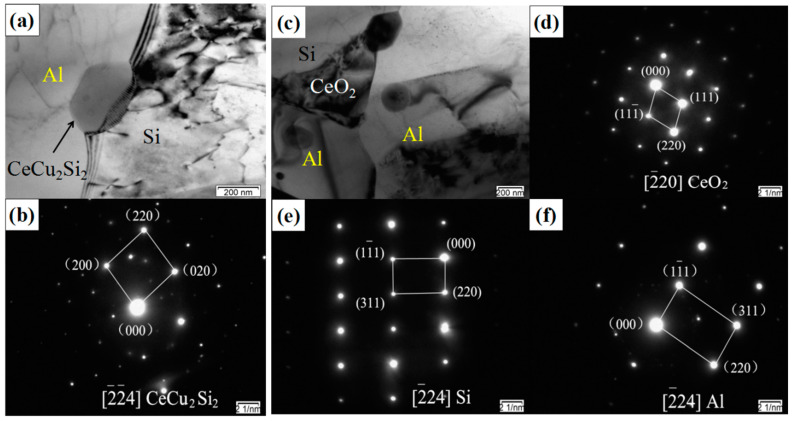
TEM images of SiCp/Al-Si composites with 0.2 vol% CeO_2_ content: (**a**) the precipitated phase CeCu_2_Si_2_; (**b**) diffraction patterns of CeCu_2_Si_2_; (**c**) CeO_2_-Si and CeO_2_-Al interface; (**d**) diffraction patterns of CeO_2_; (**e**) diffraction patterns of the precipitated Si; (**f**) diffraction patterns of Al.

Figure 6 shows the TEM images and corresponding selected-area electron diffraction patterns of the SiCp/Al-Si composites with CeO_2_. It can be seen from Figure 6 that CeO_2_ existed at the grain boundaries of matrix, and a new phase CeCu_2_Si_2_ was formed.

### 3.3. Effect of CeO_2_ Content on Tensile Properties of SiCp/Al-Si Composites

**Figure 7:**
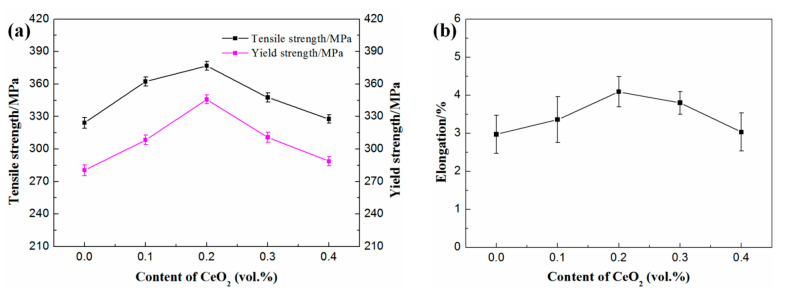
Tensile properties of SiCp/Al-Si composites with different CeO_2_ contents: (**a**) tensile strength and yield strength; (**b**) elongation.

Figure 7 shows the tensile properties curve of composites with different CeO_2_ contents. With the increase of CeO_2_ content, the tensile strength, yield strength, and elongation all showed a trend of first increasing and then decreasing. The tensile strength, yield strength, and elongation of the composites reached the peak values of 376.9 MPa, 345.8 MPa, and 4.09%, respectively, when the CeO_2_ content was 0.2 vol%, which were then increased by about 16.3%, 23.3%, and 37.7%, respectively, compared with that without CeO_2_.

On the one hand, explained from the aspect of the matrix Al-Si alloy, the relationship between the strength of Al-Si alloy and the size of Si particles can be represented as:

(1) $$\sigma \propto \frac{F_{V}}{D\left(1-F_{V}\right)}$$

in which *σ* is the strength of the alloy and *F_V_* and *D* denote the volume fraction and average diameter of Si particles, respectively. For the SiCp/Al-Si composites, it is assumed that SiC particles are equivalent to Si particles and the strength of the composites is proportional to the volume fraction of Si particles and inversely proportional to the average diameter of Si particles.

On the other hand, it can also be explained in terms of the dislocation density strengthening mechanism of the SiCp/Al-12Si composite. Dislocation density strengthening means that, when the material is cooled from the preparation or heat treatment temperature, due to the large difference in thermal expansion coefficient between the reinforcing particles and the matrix, thermal mismatch strain is generated inside the aluminum matrix, which generates a large number of dislocations, thereby improving the strength of the material. Assuming that the shape of the reinforcing particles is cubic, the increment of the yield strength caused by the increase of the dislocation density can be estimated by the following equation:

(2) $$\Delta \sigma_{d}=kG_{p}b{\left(12\frac{\Delta \alpha \Delta TV_{p}}{bd_{p}}\right)}^{1/2}$$

In Equation (2), *k* is the coefficient; *Gp* is the elastic modulus of the reinforced particles; *b* is the Burgers vector; Δ*α* is the difference in thermal expansion coefficient between the matrix and the reinforced particles; Δ*T* is the temperature difference; *dp* is the size of the reinforced particles. It is also assumed that the Si particles are equivalent to SiC particles, and it can be seen from Equation (2) that the increment in the yield strength of the composites has a negative correlation with the reinforced particle size *dp*.

Therefore, from the above two aspects, it can be concluded that the strength of the composites has a negative correlation with the Si phase particles size, that is, the smaller the Si phase particle size, the higher the strength of the composites. This can be verified from the change law of tensile strength and yield strength in Figure 7a. When the CeO_2_ content was 0.2 vol% (the average diameter of Si particles is the smallest), the tensile properties of the composites were the best.

The strengthening mechanism of composites can be analyzed from the following three aspects. Firstly, the distribution uniformity of the Si particle in the composites was considered. The existence of Si particles made the dislocation movement difficult, which can prevent the plastic deformation of the Al matrix, thereby increasing the strength. The hindering effect of Si particles on dislocation enhanced with the increase of its distribution uniformity. At this time, the stress concentration degree decreased gradually, and the stress limit required for fracture became slow, thus improving the strength and ductility (elongation) of composites. Secondly, the size of Si particles was one of the main factors affecting the strength of composites. On the one hand, the large-size Si particles were easy to crack during the stress process due to their brittleness and became the crack initiation site for the composite fracture, thus reducing the strength and elongation of the material. On the other hand, the smaller the particle size of Si phase, under the condition of a certain Si phase content, the number of Si phase particles will increase greatly. The phase interface between Al and Si then increased greatly, and the obstacle to dislocation slip was enhanced so as to improve the strength. Finally, the CeO_2_ content also had a great influence on the strength of composites. When the volume fraction of CeO_2_ exceeded 0.2%, the agglomeration phenomenon of CeO_2_ was intensified. The internal structure of the agglomerated CeO_2_ was loose, which could not effectively bear the stress. At the same time, the ability to transfer stress was also poor. In addition, agglomerated CeO_2_ was irregular in shape, which easily became the crack initiation site in the process of plastic deformation, thus reducing the strength of the material.

### 3.4. Fracture Behavior of SiCp/Al-Si Composites

**Figure 8:**
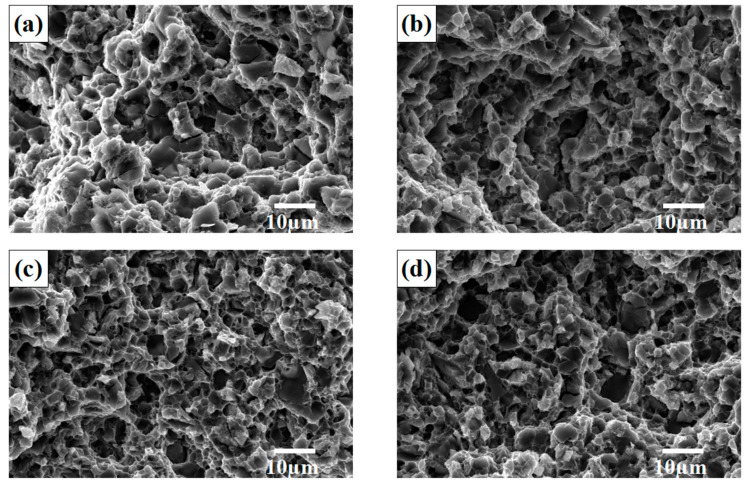

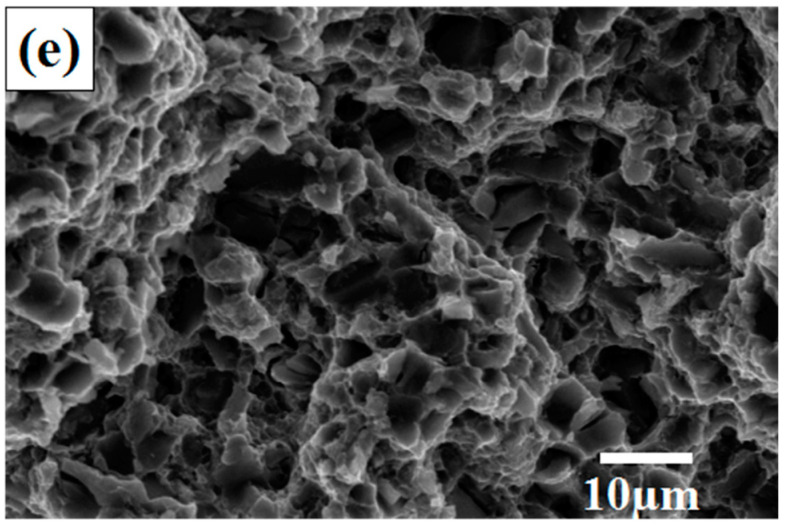
The SEM images of fracture surface of SiCp/Al-Si composites with different volume fraction of CeO_2_: (**a**) 0; (**b**) 0.1%; (**c**) 0.2%; (**d**) 0.3%; (**e**) 0.4%.

Macroscopically, there was no obvious plastic deformation of the composites before tensile fracture. From the tensile properties measured above, it can be seen that the elongation of the composites was between 2.97% and 4.09%, so the fracture form was brittle. However, from a microscopic point of view, with the change of the CeO_2_ volume fraction, its fracture behavior also had a certain difference. Figure 8 shows the SEM images of the fracture surface of the composites with different volume fractions of CeO_2_. Among them, Figure 8a shows the fracture morphology of the composites without CeO_2_. There were a large number of Si phase particles or large-size SiCp cleavage sections, cavities formed by the peeling of SiC particles and the tearing edges formed around the cavities on the fracture. In addition, there were small toughening nests generated by the fracture of the Al matrix distributed around Si and SiC. With the increase of CeO_2_ volume fraction, the section size of Si phase decreased and the number of toughening nests and tearing edges increased, reaching the peak when the CeO_2_ content is 0.2 vol%. At this time, the cross section of the Si phase particles was the smallest, and the number of toughening nests and tearing edges was the largest. Thereafter, as the content of CeO_2_ continued to elevate, the cross-section size of the Si phase particles increased, and the number of toughening nest and tearing edges reduced.

On the one hand, the fracture behavior of the tensile specimens was mainly related to the size of the Si phase particles. The cleavage section was mainly produced by the brittle fracture of Si and SiC particles, and the addition of an appropriate amount of CeO_2_ could refine the Si phase particles, so that the size of the cleavage section formed by the Si particles was reduced and the number of tearing edges and small toughening nests around the Si particles increased accordingly. On the other hand, SiC particles could be drawn, while Si particles mainly underwent cleavage fracture, which was mainly caused by the bonding strength of the interface. The average size of SiCp was 5 µm; the size was small and the internal defects were few, so the particle fracture strength was high. The interface bonding strength between SiCp and Al was less than the fracture strength of SiCp, so cracks were formed along the interface junction and then fractures occurred. The Si particles were better combined with Al, and the interface bonding strength was greater than the fracture strength of Si particles, so the Si particles had cleavage fracture. Furthermore, the fracture of the tensile specimen started from the fracture of Si particles and SiCp. As the tensile stress increased, stress concentration occurred around the reinforcement particles and the Si particles and SiCp were fractured, forming the crack source. The cracks then propagated into the Al matrix and connected to each other, which eventually lead to the occurrence of fracture behavior. There is research showing that the small Si particles have an impediment to the crack growth [24], so the refinement of Si particles can improve the fracture strength and plasticity of the material by hindering the growth of cracks. The uniform distribution of Si particles relatively reduced the stress concentration, thereby hindering fracture.

In a nutshell, the fracture mode of the 20 vol% SiCp/Al-12Si composites with rare earth addition was a mixed fracture: brittle cleavage fracture of the Si phase and a few SiC particles, the tearing of Si and SiC particles with the matrix at the matrix interface, and ductile fracture in Al matrix far away from Si and SiC particles.

### 3.5. Analysis of the Mechanism of CeO_2_ in SiCp/Al-Si Composites

As can be seen in Figure 4f, the addition of CeO_2_ significantly refined the precipitated silicon phase. During the sintering process, the Si solute was precipitated from the supersaturated solid solution Al matrix. The formation of Si phase included two stages: nucleation and growth. The higher the nucleation rate and the lower the growth rate, the smaller the size of Si particles. The high energy locations, such as grain boundaries, high-density dislocations, and second-phase particles, are preferentially heterogeneous nucleation sites for the silicon phase [25,26,27]. The nucleation rate of heterogeneous nucleation depended on the interface energy between the substrate and the nucleation phase. The nucleation resistance decreased with the decrease of interface energy, which was conducive to heterogeneous nucleation and increased the nucleation rate.

According to lattice mismatch theory [28], as the mismatch degree between substrate and nucleation phase decreases, the lattice matching degree between substrate and nucleation phase increases, so that the energy caused by lattice mismatch at the interface reduces, that is, the interface energy between substrate and nucleation phase reduces. The interface energy is the resistance to the nucleation of the silicon phase. Al, Si, and CeO_2_ have face-centered, diamond, and fluorite-type (CaF_2_) cubic structures, respectively, all of which belonging to the cubic crystal system and face-centered cubic lattice. Therefore, the one-dimensional mismatch degree can be used to reflect the nucleation effect of the substrate and CeO_2_ on the Si phase. The one-dimensional lattice mismatch degree (*δ*) can be expressed as:

(3) $$\delta =\frac{|\alpha_{s}-\alpha_{n}|}{\alpha_{n}}$$

where *α_s_* is the lattice constant of the substrate and *α_n_* is the lattice constant of the nucleation phase. From the calibrated diffraction spectrum in Figure 6, it can be seen that the lattice constants of the cubic system Al, Si, and CeO_2_ phase were 0.4050 nm, 0.5430 nm, and 0.5411 nm, respectively. Substituting into Equation (3), the one-dimensional mismatch of the Al substrate and Si phase (*δ*_1_) and the CeO_2_ substrate and Si phase (*δ*_2_) were calculated to be 0.254 and 0.0035. *δ*_2_ is much smaller than *δ*_1_, and the difference between the two is two orders of magnitude. According to the lattice mismatch degree, it can be seen that the interface between the Al substrate and Si phase was a non-coherent interface, while the interface between the CeO_2_ substrate and Si phase was a coherent interface. The interface energy of the non-coherent interface was greater than that of the coherent interface (as shown in Table 2). That is, the nucleation resistance was lower with CeO_2_ as the substrate for Si than with Al as the substrate, and the heterogeneous nucleation effect was better. Therefore, adding CeO_2_ to the SiCp/Al-Si composites, CeO_2_ can provide a large number of nucleation substrates to refine the Si particles.

The rare earth (CeO_2_) interacted with the alloying elements in the aluminum matrix to form the rare earth compounds (Figure 6a). The particles were small, which can impede the movement of dislocations and exert a certain dispersion strengthening effect. At the same time, the new phase CeCu_2_Si_2_, located at the interface of Al and Si, can improve the bonding strength between Al and Si particles.

According to Figure 6a,c, it can be seen that CeO_2_ dispersoids existed at the grain boundary or phase boundary, which significantly reduced the mobility of the interface and hindered movement of the aluminum grain boundary. In the process of sintering, hot extrusion, and heat treatment, it can hinder the growth of the aluminum grains to a certain extent and refine the grains, thereby simultaneously improving the strength and plasticity of the composites.

## 4. Conclusions

(1) The addition of an appropriate amount of CeO_2_ can refine the Si particles, reduce the agglomeration of Si phase, and improve its distribution uniformity in the SiCp/Al-Si composites. When the CeO_2_ volume fraction was 0.2%, the Si phase particle size was the smallest and the distribution uniformity was the best.
(2) The main precipitates in SiCp/Al-Si composites without CeO_2_ were Al_19_Mn_4_, Al_4_Cu_9_, Al_2_Cu, Al_5_Cu_6_Mg_2_, and a new phase of CeCu_2_Si_2_ was formed after adding CeO_2_ in the composites. CeO_2_ was mainly located at the grain boundary or phase boundary of the composites.
(3) The addition of appropriate content of CeO_2_ can improve the tensile properties of composites. When the CeO_2_ content was 0.2 vol%, the tensile properties of the composites were the best.
(4) The fracture mode of the 20 vol% SiCp/Al-12Si composites with rare earth addition is a mixed fracture: brittle cleavage fracture of the Si phase and a few SiC particles, the tearing of Si and SiC particles with the matrix at the matrix interface, and ductile fracture in the Al matrix far away from Si and SiC particles.
(5) There are three main mechanisms of CeO_2_ in SiCp/Al-Si composites. Firstly, CeO_2_ serves as the nucleation substrate of Si phase to refine Si particles; secondly, CeO_2_ reacts with the alloying elements in the aluminum matrix to form a new phase CeCu_2_Si_2_, which can exert a certain dispersion strengthening effect and improve the bonding strength between Al and Si particles; thirdly, is the pinning effect of CeO_2_ and CeCu_2_Si_2_ particles on grain boundaries or phase boundaries to refine aluminum grains.

## Figures and Tables

**Table 1:** Chemical compositions of Al-12Si alloy (wt%).

Component	Si	Cu	Mg	Mn	Al
Content	12.0	1.4	0.7	0.6	Bal.

**Table 2:** The relationship among interface structure, lattice mismatch degree, and interface energy.

Interface Structure	Lattice Mismatch Degree	Interface Energy
Coherent Interface	*δ* ≤ 0.05	0.1 J/m^2^
Semi-coherent Interface	0.05 ≤ *δ* ≤ 0.25	0.5 J/m^2^
Non-coherent Interface	*δ* ≥ 0.25	1.0 J/m^2^

## Data Availability

All data generated or analysed during this study are included in this article.
